# A systematic review of insecticide resistance in *Aedes aegypti* (Diptera: Culicidae) and implications for dengue control in Indonesia

**DOI:** 10.14202/vetworld.2025.658-672

**Published:** 2025-03-18

**Authors:** Muhammad Rasyid Ridha, Ririh Yudhastuti, Hari Basuki Notobroto, Muhammad Choirul Hidajat, Khuliyah Candraning Diyanah, Babucarr Jassey, Ghina Maulida Rahmah

**Affiliations:** 1 Doctorate Degree Program in Public Health, Faculty of Public Health, Universitas Airlangga, Surabaya, Indonesia; 2 Research Center for Public Health and Nutrition, National Research and Innovation Agency Republic of Indonesia, Jakarta, Indonesia; 3 Department of Environmental Health, Faculty of Public Health, Universitas Airlangga, Surabaya, Indonesia; 4 Department of Epidemiology, Biostatistics, Population Studies and Health Promotion, Faculty of Public Health, Universitas Airlangga, Surabaya, Indonesia; 5 Department of Public Health Services, Ministry of Health, Quadrangle, Banjul, The Gambia, 00220, West Africa; 6 Statistics Study Program, Faculty of Mathematics and Natural Sciences, University of Lambung Mangkurat, Banjarbaru, Indonesia

**Keywords:** *Aedes aegypti*, dengue fever, Indonesia, insecticide resistance, knockdown resistance mutations, vector control

## Abstract

**Background and Aim:**

Dengue fever, primarily transmitted by *Aedes aegypti*, remains a critical public health challenge in Indonesia, with periodic outbreaks exacerbated by widespread insecticide resistance. Resistance to organophosphates and pyrethroids limits vector control efforts, necessitating updated insights into resistance patterns and their genetic underpinnings. This study aimed to evaluate and map insecticide resistance and associated genetic mutations in *Ae. aegypti* across Indonesia, providing actionable insights for vector management strategies.

**Materials and Methods:**

This systematic review adheres to Preferred Reporting Items for Systematic reviews and Meta-Analyses guidelines, encompassing studies from 2010 to 2023 identified through PubMed, Scopus, EBSCOhost, and Embase. Keywords targeted *Ae. aegypti*, insecticide classes, resistance, and Indonesian regions. Inclusion criteria focused on field-derived populations subjected to World Health Organization bioassays for organophosphates (malathion and temefos) and pyrethroids (cypermethrin, deltamethrin, etc.), alongside analyses of knockdown resistance (*kdr*) mutations in the voltage-gated sodium channel (*Vgsc*) and acetylcholinesterase-1 (*Ace-1*) genes. Data synthesis included resistance trends, spatial mapping, and allele frequency analyses.

**Results:**

Resistance to malathion and temefos is extensive, with sporadic susceptibility in specific districts. Pyrethroid resistance is pervasive, particularly for cypermethrin and lambda-cyhalothrin, with deltamethrin exhibiting isolated susceptibility. Genetic analyses reveal *Vgsc* mutations (V1016G, F1534C) as key drivers of pyrethroid resistance, while *Ace-1* mutations remain unreported. The evolution of resistance correlates with indiscriminate insecticide usage, urbanization, and climatic factors.

**Conclusion:**

The growing prevalence of insecticide resistance in *Ae. aegypti* underscores the urgent need for integrated vector management strategies. These should incorporate insecticide rotation, resistance monitoring, and community engagement to mitigate resistance and support sustainable dengue control efforts in Indonesia.

## INTRODUCTION

Dengue hemorrhagic fever (DHF) – caused by the dengue virus, namely, dengue-1, dengue-2, dengue-3, and dengue-4 – is a public health issue in Indonesia. *Aedes aegypti* is the primary vector of this illness [1]. Increases in cases frequently occur at several locations, particularly at the beginning of and throughout the rainy season, when environmental conditions facilitate the proliferation of mosquito vectors. *Ae. aegypti* mosquitoes are active throughout the day and tend to nest in close proximity to bathtubs, trashcans, and other water containers found in and around houses. Efficient vector control measures are crucial for halting the transmission of dengue [2, 3].

Insecticidal fumigation (fogging) is one of the most common control methods for disease spread. This method employs a fogging machine to disperse small insecticide particles into the air to kill adult mosquitoes, which are responsible for causing the disease, while reducing the possibility of transmission of the dengue virus [4, 5]. In addition, larvicides are combined with chemical insecticides, such as temefos, by sowing them into water reservoirs to kill the mosquitoes at the larval stage. However, the widespread and repeated use of insecticides has resulted in insecticide resistance in vector mosquitoes. This resistance hinders dengue control efforts because mosquitoes develop defense mechanisms against these chemicals, and previously effective insecticides do not have the desired effect [6, 7]. The indiscriminate use of insecticides has caused significant toxic environmental impacts, including cumulative effects on ecosystems. Insecticides accumulated in soil, water, and air can damage various trophic layers in the food chain, ranging from low-level organisms such as non-target insects and plankton to top-level predators through bioaccumulation and biomagnification processes [8]. In addition, backfire effects on humans have also been widely demonstrated, such as health disorders caused by exposure to insecticide residues, including the risk of neurological disorders, hormonal imbalances, and increased cancer incidence. Studies have shown that the uncontrolled use of insecticides also contributes to the decline of beneficial insect populations, such as pollinators, which disrupt ecosystem balance and agricultural productivity [9–11]. Therefore, proper management and monitoring of insecticide use is essential to prevent adverse impacts on the environment and human health. Several factors can cause insecticide resistance in *Ae. aegypti* mosquitoes. Excessive use of insecticides, excessive fumigation, and selection pressure on mosquito populations can increase the selection pressure. In addition, resistance growth can be influenced by environmental factors such as climate [12]. Insecticide resistance has a major impact on mosquito control. The reduced effectiveness of fogging and larvicides also escalates the risk of increased vector mosquito populations; hence, dengue control programs may become less effective, and disease cases may continue to increase. The geographic and demographic characteristics of Indonesia make it difficult to control dengue fever [13, 14]. Environmental diversity, limited resource availability, and high population mobility are additional challenges to managing insecticide resistance at the local level [15].

Therefore, a thorough understanding of insecticide resistance and its mechanisms is important in various regions of Indonesia. These changes have allowed health experts and researchers to assess the effectiveness of insecticides, create better resistance management plans, and adapt vector control programs to local circumstances. Therefore, it is important to involve the community in controlling insecticide resistance [16, 17]. This can be achieved by educating the public on the dangers of uncontrolled insecticide use, encouraging dengue prevention habits at the household level, and actively participating in vector control programs. Managing insecticide resistance requires a comprehensive approach involving collaboration between researchers, health institutions, and interested parties [17–19].

Updated research on insecticide resistance in Indonesia should be conducted to determine the distribution of various types of insecticides. This study aimed to estimate and map the prevalence of insecticide resistance, as well as knockdown resistance (*kdr*) mutations, in *Ae. aegypti* in Indonesia. We examined three common *kdr* mutations (F1534C, V1016G, and S989P) in *Ae. aegypti*. The analysis of *A. albopictus* using World Health Organization (WHO) and *kdr* techniques was not conducted due to the limited sample size. Understanding the current status and distribution of vector control is expected to help plan vector management in Indonesia.

## MATERIALS AND METHODS

### Ethical approval

This systematic review was conducted in accordance with the Preferred Reporting Items for Systematic Reviews and Meta-Analyses statement [20].

### Study period and location

The search period began in December 2023 through June 2024 and data analysis began in July through August 2024. Data were processed at the Department of Environmental Health, Faculty of Public Health, Universitas Airlangga.

### Search strategy

We reviewed published studies on the prevalence of insecticide resistance in *Ae. aegypti*. Four databases – PubMed, Scopus, EBSCOhost, and Embase were used for the search. Search keywords were created by combining “*Aedes aegypti*” OR “Organophosphates” OR “Pyrethroids” AND “Resistance” OR “Indonesia.” The article you are looking for is in English or Indonesian.

### Study selection process

**Figure 1:**
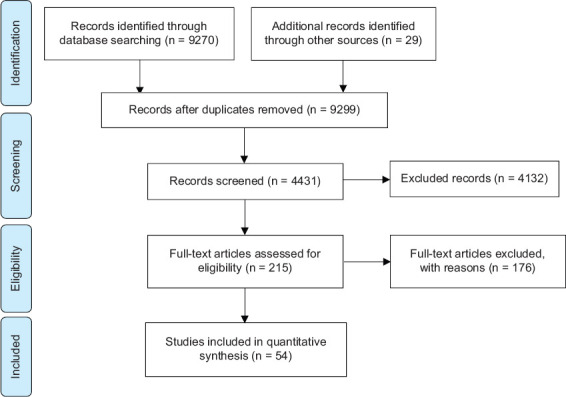
Preferred Reporting Items for Systematic Reviews: Flow diagram illustrating selection methodology.

The collected articles were selected by the authors MRR, KC, and BJ. The articles were then verified and carefully reviewed by RY, and HB considering the methods and results and analysis used. Articles were then manually tabulated by creating detailed tables on location, year, mosquito species, resistance status, method of resistance testing, type and class of insecticide, and resistance rate if any. The results were then checked again with MCH for final verification. Inclusion criteria were studies on resistance testing in *Ae. aegypti* in Indonesia. The articles included information on sample size and positive results. The use of insecticide bioassays using the WHO Tube test method with organophosphate (malathion 5%, malathion 5%, temefos) and pyrethroid (cypermethrin, alpha-cypermethrin, lambda-cyhalothrin, and deltamethrin) groups and investigating the presence of *kdr* mutations in *Ae. aegypti* at larval and adult stages were included in the criteria of this study. Exclusion criteria were research records marked before 2010, irrelevant research subjects, no prevalence information, no report on resistance test, outside Indonesia, and unclear results. In total, 9,720 scientific publications were identified. After removing records after duplicates were removed, records were screened, and full-text articles were assessed for eligibility, studies included in the quantitative synthesis were 54 articles (Figure 1).

### Data extraction and synthesis

#### Study eligibility

Articles were extracted by creating an agreed table, namely, location, year, mosquito species, resistance status, method of resistance testing, type and class of insecticide, and resistance rate. Data were synthesized in a narrative way by organizing studies based on relevant characteristics, summarizing the main findings, identifying patterns and differences in study results, and conducting analysis and conclusions.

### Bias assessment

We used the JBI Critical Appraisal Tools, developed by the Joanna Briggs Institute, to avoid bias. The assessment was conducted independently by two researchers using a list of questions relevant to each type of study included, such as experimental, observational, or cross-sectional studies. Important aspects such as completeness of research methods, clarity of inclusion and exclusion criteria, validity of measurements, potential selection bias, and transparency in reporting results. If there are differences, a third researcher was involved as an arbitrator. This process aims to minimize subjective bias and ensure that only studies with robust and valid methodologies are used in the analysis. In addition, we also considered possible publication bias by evaluating gray literature.

### Statistical analysis

Data for each article were synthesized using Humata AI (https://app.humata.ai/) to review each eligible article, then data were transferred to Microsoft Excel 2019 (Microsoft, Washington, USA) to tabulate location, year, mosquito species, resistance status, resistance testing method, insecticide type and class, and resistance level. Location, frequency of genetic mutations, and regional distribution and records. Spatial analysis in thematic maps was conducted using ArcGIS Desktop (ArcMap) version 10.8.2 to map the distribution of resistance across districts in Indonesia. [21].

## RESULTS

### Dengue cases in Indonesia

**Figure 2:**
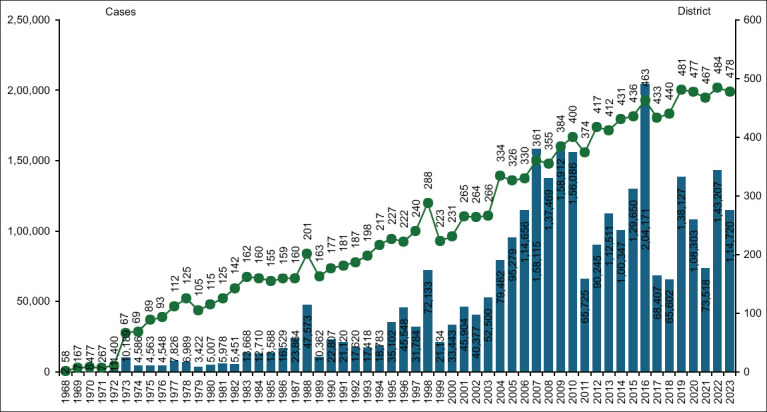
Number of dengue cases and number of dengue-infected districts annually from 1968 to 2023 [Source: Ministry of Health, Indonesia].

**Figure 3:**
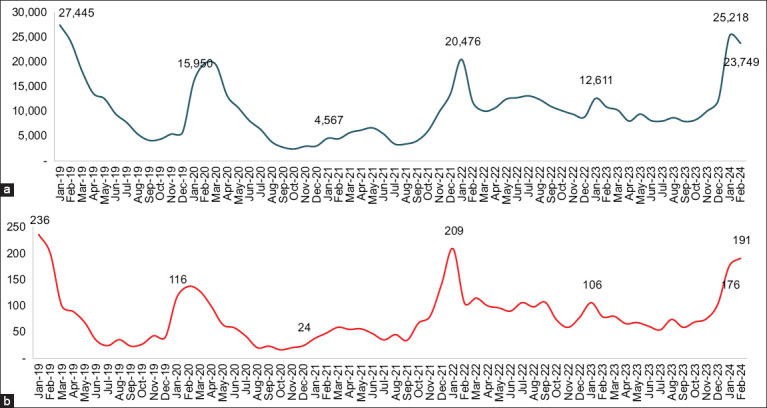
Dengue trends 2019–2024 (a) cases and (b) fatalities [Source: The map was generated using ArcGIS 10.8.2].

Figure 2 shows the trend in the number of DHF cases and the number of districts reporting cases each year in Indonesia from 1968 to 2023. The graph shows a significant spike in cases in 2016, followed by another increase in 2019 and 2023. The overall trend shows annual fluctuations with a tendency for cases to increase over time. The graph also depicts a consistent increase in districts reporting DHF cases, indicating the widespread spread of the disease across Indonesia. Figure 3 consists of two panels depicting the trend of DHF in Indonesia from 2019 to 2024. Figure 3a shows the number of dengue cases recorded annually. The highest peak occurred in 2020, followed by a decrease in cases in the following year. However, although the number of cases decreased, there was a rebound in certain years, displaying an inconsistent pattern. Figure 3b shows the number of DHF deaths over the same period. Unlike the number of cases, the trend of deaths was relatively stable or slightly decreased each year. This graph shows the difference in the pattern between the fluctuating number of cases and the more stable number of deaths over time.

### Spread of resistance in Indonesia

**Figure 4:**
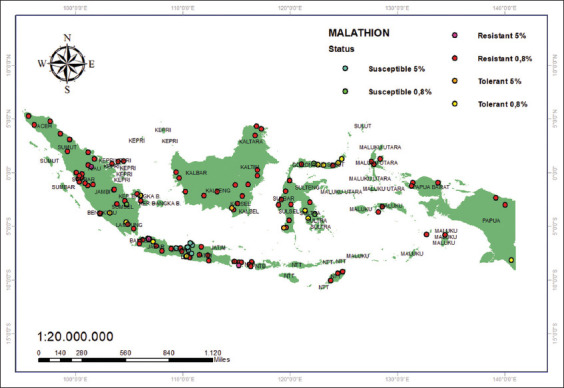
Malathion resistance map (0.8% and 5%) [Source: The map was generated using ArcGIS 10.8.2].

Malathion resistance is widespread in Indonesia [22–32], and a few districts still contain vulnerable populations, namely, Kudus [23], Gorontalo, and several districts in Southeast Sulawesi (Figure 4) [33]. The maps presented here focus on diagnostic malathion doses of 0.8% and 5%.

**Figure 5:**
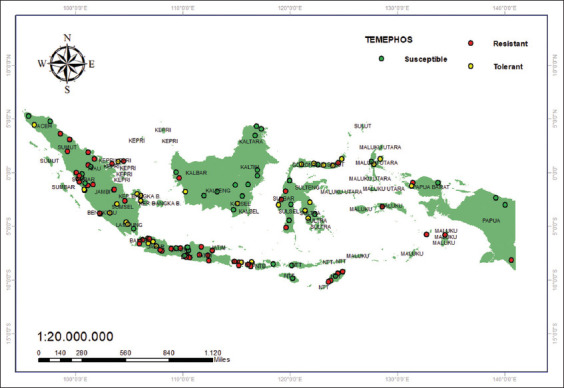
Temefos resistance status (0.02%) [Source: The map was generated using ArcGIS 10.8.2].

The use of temefos is well established in Indonesia; most districts and cities contain resistant populations, with a primary distribution in Sumatra and Java. As many as 30 (29%) districts/cities have a tolerant status, while 22 (22%) districts still contain vulnerable populations. Districts/cities with vulnerable status include Banda Aceh City, Lhoksemauwe City, Metro City, Kotawaringin Timur, Kotawaringin Barat, Palangkaraya City, Hulu Sungai Selatan, Tabalong, Mempawah, Bontang, Samarinda, Balikpapan, Palopo, Makassar, Palu, Tarakan, Nunukan, Bulungan, Ternate, Sorong, Jayapura, and Jayapura City (Figure 5) [33–40].

**Figure 6:**
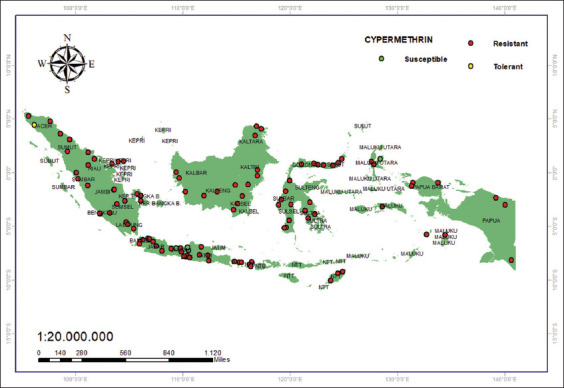
Resistance status of cypermethrin (0.05%) [Source: The map was generated using ArcGIS 10.8.2].

Figure 6 illustrates the cypermethrin resistance status. Resistance to cypermethrin has been reported in almost all districts of Borneo, Sumatra, and Sulawesi [33]; except in Java, where no resistant mosquito populations were found in Semarang, Pemalang, and Tegal [41].

**Figure 7:**
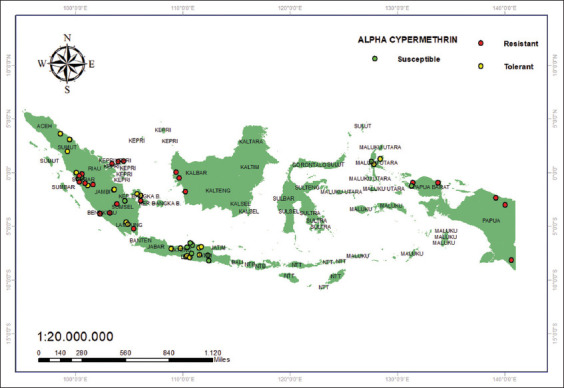
Alpha-cypermethrin 0.025% resistance status [Source: The map was generated using ArcGIS 10.8.2].

The alpha-cypermethrin resistance status varies across Indonesia (Figure 7); however, most districts/cities contain resistant populations. The districts still reported to contain vulnerable populations include Semarang, Kudus, Surakarta, Jepara [41], Batusangkar [42], and Pariaman in West Sumatra [43].

**Figure 8:**
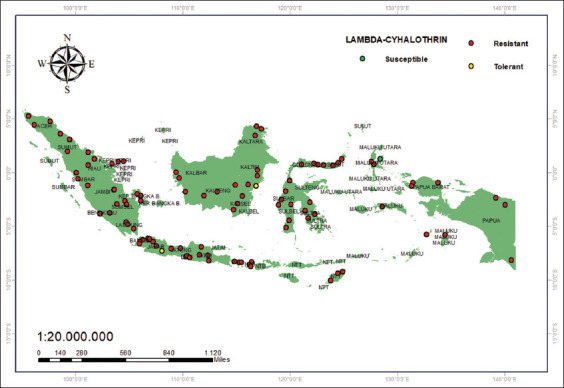
Resistance status of lambda-cyhalothrin (0.03%) [Source: The map was generated using ArcGIS 10.8.2].

**Figure 9:**
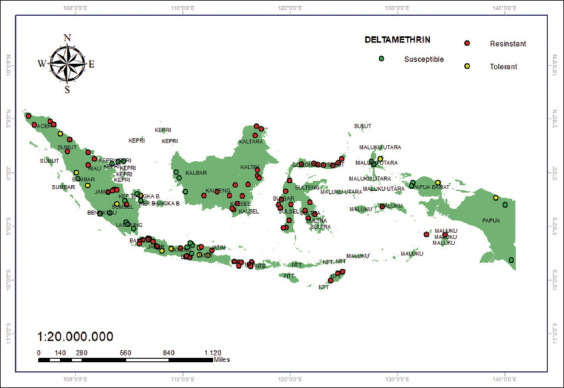
Resistance of deltamethrin to 0.025% [Source: The map was generated using ArcGIS 10.8.2].

The resistance status of mosquitoes against lambda-cyhalothrin in Indonesia is predominantly resistant, excluding Balikpapan on the island of Kalimantan, where the status is believed to be tolerant (Figure 8) [33, 44]. Meanwhile, mosquitos were found to be susceptible to the insecticide deltamethrin; specifically, 22 districts/cities [33, 41] were identified as vulnerable: Padang, Bengkulu, Lubuk Linggau, Rejang Lebong, Metro, Tanjung Karang, Pringsewu, Batam, Tanjungpinang, Tanjung Balai, Pangkal Pinang, West Bangka, Mempawah, Kubu Raya, Ketapang, Merauke, Jayapura, Sorong, Sorong, Ternate, and Tidore (Figure 9).

### Detection of *kdr* mutations in voltage-gated sodium channel *(Vgsc)* and acetylcholinesterase-1 *(Ace-1)* genes

Table 1 [23, 25–29, 31, 32, 41–43, 45–55] presents the genetic changes linked to pesticide resistance in the *Ae. aegypti* mosquito population in Indonesia. This table contains information on mutations in *Vgsc*, which is linked to mutations in the *kdr* and *Ace-1* genes. The prevalence of *kdr* mutations, specifically V1016G and F1534C, in different mosquito populations from different regions in Indonesia, including Kuningan, Padang, Samarinda, Pontianak, Denpasar, and Mataram, where its allele frequency reached 1.00. In contrast, no V1016G was detected in Dompu and West Manggarai, indicating susceptibility in these populations. The F1534C mutation has been reported in areas such as Banjarmasin, Makassar, and Muaro, with varying frequencies, contributing to different levels of resistance. Cities such as Jakarta, Yogyakarta, and Surabaya showed both mutations, while Central Java areas such as Semarang and Tegal also showed a significant frequency of V1016G (91.2%) alongside S989P and F1534C. In Palu, the V1016G mutation was present, but no mutations in the *Ace-1* gene were detected. The table also includes information on the prevalence of resistance alleles at each location, demonstrating variations in the extent of resistance observed in different regions.

**Table 1:** Detection of *Kdr* mutations in *Vgsc* and *Ace-1* genes of *Ae. aegypti* mosquitoes in Indonesia.

No.	Location	Mutation	Year of publication	Frequency of Resistant Alleles	Notes	References
1	Holy	S989P, V1016G	2016	S989P: 25%; V1016G: 91.2%, F1534C: Not detected	Allele frequencies reflect the extent of genetic diversity that contributes to resistance to pyrethroid pesticides.	[23]
2	Jepara	S989P, V1016G, and F1534C	2016	S989P: 25%, V1016G: 91.2%, F1534C: 3%	Allele frequencies reflect the extent of genetic diversity that contributes to resistance to pyrethroid pesticides.	[23]
3	Surakarta	S989P, V1016G, and F1534C	2016	S989P: 25%, V1016G: 91.2%, F1534C: 3%	Allele frequencies reflect the extent of genetic diversity that contributes to resistance to pyrethroid pesticides.	[23]
4	Banjarmasin	V1016G, F1534C	2018	V1016G: G: 0.545, V: 0.455	The V1016G mutation exhibited a notable correlation with the permethrin resistance phenotype, whereas the F1534C mutation was not significantly associated with this resistance.	[25]
				F1534C: C: 0.302, F: 0.698		
5	Jakarta	V1016G, F1534C	2017	V1016G: Homozygous GG: 0.40%, Heterozygous VG: 0.48%; total G allele frequency: 0.64%	High resistance to permethrin; V1016G was significant regarding resistance, while F1534C was not significant.	[26]
				F1534C: Homozygous CC: 0.03%, Heterozygous FC: 0.033%; total C allele frequency: 0.20%		
6	Denpasar, Bali	F1534C, S989P, and V1016G	2017	F1534C: Homozygous CC: 0.21, Heterozygous FC: 0.25 (resistant phenotype)	The *Ae. aegypti* population in Denpasar exhibited high resistance to permethrin. The frequency of F1534C, S989P, and V1016G mutations was significantly associated with resistance.	[27]
				S989P: Half of the permethrin-resistant phenotype with *P* allele frequency		
				V1016G: OR: 2.49		
7	Palu, Central Sulawesi, Indonesia	V1016G, *Ace-1* (G119)	2019	V1016G: Found in Palu. G119 (*Ace-1*): Found in Palu.	The V1016G mutation in *Vgsc* gene was detected in Palu, indicating resistance to pyrethroid insecticides. There were no mutations in *Ace-1* in Palu. The G119 wild-type allele suggests that organophosphate resistance likely occurs through other mechanisms; in particular, metabolic resistance.	[28]
8	Surabaya	V1016G, F1534C, and *AChE*	2021, 2021	Not mentioned	Research indicates mutations in the *Vgsc* and *AChE* genes that contribute to insecticide resistance. Understanding genetic mutations is important for more effective vector control.	[29]
9	Yogyakarta	F1565C, V1023G, and S996P	2018	F1565C: Very low frequency; only one individual was homozygous (1565C/1565C) among 151 samples. V1023G: 83% homozygous (GG) | S996P: 17% homozygous	The *Aedes aegypti* population in Yogyakarta exhibited high resistance to deltamethrin and permethrin. The V1023G and S996P mutations were associated with resistance, whereas the F1565C mutation was not.	[31]
			2021, 2024			[47]
						[48]
				V1023G: Homozygotes 1023G/1023G dominate at 58% in Season 1		[49]
				S996P: homozygous frequency of 9.27%		[50]
10	Jambi City	F1534C, V1016G	2022	It was not specifically stated, but the V1016G mutation was more common than the F1534C mutation.	Overall, 30% of the mosquitoes exhibited heterozygous F1534C mutations, whereas none exhibited homozygous mutations. Regarding the V1016G mutation, 59.1% of the samples exhibited this mutation.	[32]
11	Batang Hari Regency, Batang, Thailand	F1534C, V1016G	2022	It was not specifically stated, but the V1016G mutation was more common than the F1534C mutation.	Overall, 40% of the mosquitoes exhibited the heterozygous F1534C mutation; none exhibited the homozygous mutation. The V1016G mutation was also more prevalent than the F1534C mutation.	[32]
12	Semarang, Semarang Regency; Tegal, Pemalang Regency	S989P, V1016G	2023	S989P: 57.9% | V1016G: 78.9%	The S989P mutation was detected in 57.9% of samples, whereas the V1016G mutation was present in 78.9% of samples. There are no known mutations in *Ace-1*. *Vgsc* mutations are a prevalent route to resistance to pesticides in certain mosquito populations.	[41]
13	Padang, West Sumatra	VGSC (T506T), Ace-1 (G119S, T506T)	2019	VGSC (T506T): TT (65.21%), TA (26.08%), AA (8.69%) Ace-1: G119S (Not detected), T506T: TT (65.21%), TA (26.08%), AA (8.69%)	In Padang, VGSC mutations at location T506T were detected with allele frequencies of TT (65.21%), TA (26.08%), and AA (8.69%). In the Ace-1 gene, there was no mutation at the G119S location, but a new mutation was found at T506T with the same frequency as VGSC.	[42]
14	West Sumatra	F1534C and other variants	2018	Not mentioned	Multiple *kdr* mutations were observed in the *Ae. aegypti* population.	[43, 45]
15	Magelang City, Central Java.	S989P, V1016G		Not mentioned	Resistance to pyrethroid insecticides, particularly permethrin, was linked to mutations in the *Vgsc* IIS6 gene.	[45]
16	Kanagarian Salido, Pesisir Selatan Regency, West Sumatra, Indonesia	No resistance -related mutations were found in the *Ace-1* gene; a point mutation at codon 506 was found	2022	Not mentioned	A high level of resistance to temefos was observed, resulting in a mortality rate of 91.67%.	[46]
17	Makassar	V1016G, F1534C	2020	V1016G: G: 0.58% (resistant group), G: 0.45% (susceptible group)	The *Ae. aegypti* population in Makassar exhibited resistance to permethrin.	[51]
				F1534C: C: 0.23% (resistant group)		
18	Kuningan	V1016G	2019	V1016G allele frequency: 1.00	The V1016G mutation was the most dominant genotype in Kuningan.	[52]
19	Padang	V1016G	2019	V1016G allele frequency: 1.00	The V1016G mutation was the most dominant genotype in Padang.	[52]
20	Samarinda	V1016G	2019	V1016G allele frequency: 1.00	The V1016G mutation was the most dominant genotype in Samarinda.	[52]
21	Pontianak	V1016G	2019	V1016G allele frequency: 1.00	The V1016G mutation was the most dominant genotype in Pontianak.	[52]
22	Denpasar	V1016G	2019	V1016G allele frequency: 1.00	The V1016G mutation was the most dominant genotype in Denpasar.	[52]
23	Mataram	V1016G	2019	V1016G allele frequency: 1.00	The V1016G mutation was the most dominant genotype in Mataram.	[52]
24	Dompu	V1016G	2019	V1016G allele frequency: 0.00	The V1016G mutation was not detected in Dompu.	[52]
25	West Manggarai	V1016G	2019	V1016G allele frequency: 0.00	The V1016G mutation was not detected in West Manggarai.	[52]
26	East Sumba	V1016G	2019	V1016G allele frequency: 0.40	The V1016G mutation was detected in East Sumba.	[52]
27	Semarang, Central Java.	S989P, V1016G, and F1534C	2021	S989P: 25%, V1016G: 91.2%, F1534C: 3%	Allele frequencies reflect the extent of genetic diversity that contributes to resistance to pyrethroid pesticides.	[53]
28	Muaro, Jambi Regency, country office	F1534C, V1016G	2021	F1534C: C: 0.225, F: 0.775|V1016G: G: 0.45, V: 0.55	Overall, 35% of mosquitoes carried the heterozygous F1534C mutation, whereas 25% carried the homozygous mutation. The V1016G mutation was more common, with 40% of the mosquitoes exhibiting the heterozygous mutation.	[54]
29	Palembang	Val1016Ile	2012	No specifics were mentioned.	A point mutation in the *Vgsc* gene at the Val1016Ile position was identified as a marker of resistance to synthetic pyrethroid insecticides. No mutations were detected at the Val1016Gly position.	[55]

*Kdr*=Knockdown resistance, *Vgsc*=Voltage-gated sodium channel, *Ace-1*=Acetylcholinesterase-1, *Ae. aegypti*=*Aedes aegypti*

## DISCUSSION

Cumulatively, the number of dengue cases has been increasing annually, indicating that the dengue virus is increasingly spreading across various regions. This increase is likely influenced by various factors, such as socioeconomics [56], the environment (including climate change [57]), urbanization [58], and resistance of the *Ae. aegypti* mosquito to insecticides [24]. The number of districts reporting dengue cases has also increased, indicating that an increasing number of areas are affected by the virus. In addition, the graph displays peak cases in certain years, which could be related to the dengue cycle phenomenon [59] or major outbreaks [60]. Although there are certain periods when dengue cases decrease, the overall trend still demonstrates an increase in cases over time. This transient decrease may only indicate temporary management of the situation; a subsequent increase in the following years indicates the necessity for more efficient and enduring management methods [61], considering ecological factors such as higher rainfall, temperature, and humidity boost mosquito breeding while urbanization creates more breeding sites [62]. Epidemiologically, population mobility spreads the virus to new areas, and factors such as limited public awareness, weak vector control, and insecticide resistance further fuel transmission [63]. These combined factors make dengue management more challenging, emphasizing the need for sustainable dengue control strategies.

Between 2019 and 2024, dengue fever cases varied, with the highest number observed in 2020. This increase can be attributed to a surge in the number of *Ae. aegypti* mosquitoes, which are the primary carriers of dengue fever. Climate change and other environmental conditions that facilitate mosquito growth likely contributed to this increase. Following the peak in 2020, a subsequent decline in cases was observed in subsequent years; however, this decline was not uniform, and an increase in cases was still periodically observed.

Meanwhile, fatality trends (number of deaths) demonstrated that although the number of dengue fever cases fluctuates, the fatality rate tends to remain stable or decrease slightly from year to year. The observed reduction in fatality rates may reflect improvements in the quality of health services and management of dengue fever cases in Indonesia [64]. In addition, this approach can increase public awareness regarding the prevention and early treatment of dengue fever symptoms [53, 65]

The development of insecticide resistance in *Ae. aegypti* has emerged as a major obstacle to the management of dengue infections in Indonesia. Insecticides are widely used as the primary means of controlling mosquito populations. This includes implementing government-led fogging initiatives, applying insecticides in the agricultural sector and industrial settings (such as hotels and corporations), and using them at the household level [66]. Excessive and unregulated use has resulted in the emergence of resistance in the *Ae. aegypti* mosquito, which directly contributes to an increase in dengue cases [66–68]. Mosquitoes that are insecticide-resistant can survive despite exposure, minimizing the effectiveness of mosquito control programs and potentially causing a wider spread of dengue.

The development of insecticide resistance in *Ae. aegypti* mosquito populations in Indonesia is strongly linked to the extensive use of pesticides across multiple sectors. The use of insecticides in agriculture can lead to environmental contamination, which interacts with mosquito populations, thereby enhancing their adaptability and the development of resistance [69]. In industrial sectors – such as hotels and companies – as well as in households, insecticides are used as part of hygiene management to control insect populations [70]. This widespread and repeated use of insecticides strengthens the selection pressure on mosquitoes and promotes the development of resistance [71].

In Indonesia, insecticides from organophosphate and pyrethroid groups are commonly used in various mosquito control applications [32]; organophosphates, such as malathion and temefos, are widely used in mosquito larval control programs [72]. Given the reported resistance to malathion, the use of insecticides for fogging currently requires more insecticides containing active pyrethroid ingredients. Organophosphate groups have been widely used in Indonesia for decades until 2000. After 2000, the insecticides used were mainly from the pyrethroid class. Pyrethroids are used in dengue vector control programs owing to their low prices; thus, exposure to pyrethroid insecticides is widespread with the use of household mosquito repellents. Mosquito coils, the most popular household insecticide, are widely used because they are inexpensive and simple to purchase, costing Rp 5.000 (USD 0.35) weekly. Almost all household mosquito coils in Indonesia contain active pyrethroids [73].

Malathion was generally used in fogging programs for decades until the 2000s, whereas temefos was used as a larvicide in water sources. Pyrethroids – including cypermethrin, alpha-cypermethrin, lambda-cyhalothrin, and deltamethrin – are widely used in fogging programs, agriculture, industry, and households because they effectively kill adult mosquitoes [74].

Resistance to malathion and some insecticides from the pyrethroid group has been observed, indicating that existing control approaches must be adapted [27]. This resistance reduces the effectiveness of existing insecticides; therefore, a more comprehensive control strategy is needed, including the rotation of insecticide use, regular monitoring of resistance, and a combination of other control methods – such as environmental management and public education – to reduce the dependence on insecticides [71]. Thus, a holistic and sustainable approach to dengue control is required in Indonesia.

Genetic alterations in mosquito populations are closely associated with the development of insecticide resistance. Resistance is primarily caused by mutations in the *Vgsc* gene, which is referred to as *kdr* [75]. The *kdr* mutation alters the binding site of pesticide molecules belonging to the pyrethroid and dichlorodiphenyltrichloroethane families, resulting in the decreased efficacy of these insecticides in managing mosquito populations. Resistance can also be linked to mutations in the *Ace-1* gene, resulting in resistance to insecticides belonging to organophosphate and carbamate groups, such as malathion and temefos [76]. The detection of *kdr* mutations in the *Vgsc* and *Ace-1* genes revealed the distribution and frequency of resistance alleles in various regions. *Kdr* mutations commonly found in Indonesia include the V1016G and F1534C mutations, which contribute to pyrethroid resistance [23, 24, 27, 32]. Mutations in *Ace-1*, such as the G119S mutation, confer resistance to organophosphates, have not been reported in Indonesia. This is likely due to the limited research on the *Ace-1* gene mutation in *Ae. aegypti* mosquitoes in Indonesia [45, 46, 76]. These data indicate that resistance against the pyrethroid group that occurs in various mosquito populations in Indonesia has a strong genetic basis, which has implications for mosquito control in this region.

Understanding the occurrence and prevalence of *kdr* and *Ace-1* mutations is critical for developing efficient vector control strategies. In instances where there is a significant amount of resistance caused by *kdr* mutations in some regions, it may be necessary to decrease the use of pyrethroids or to substitute them with an insecticide from a different category that is not influenced by the mutation. Research on *Ace-1* mutations in *Ae. aegypti* associated with carbamate and organophosphate insecticide resistance is scarce worldwide. In the Namakkal region of India, Muthusamy discovered a mutation in the G119S codon of *Ae. aegypti* mosquitoes [77]. Although carbamates and organophosphates have been used for >20 years, Indonesia and Southeast Asia have not reported G119S codon mutations.

By combining genetic data on resistance with information on insecticide use and trends in dengue cases, vector control strategies can be adjusted to reduce the risk of more severe resistance and increase success in reducing dengue fever incidence in Indonesia.

## CONCLUSION

Indonesia faces significant challenges in managing dengue fever due to the widespread resistance of *Ae. aegypti* mosquitoes to commonly used insecticides. Resistance to organophosphates, such as malathion and temefos, and pyrethroids, including cypermethrin, alpha-cypermethrin, and lambda-cyhalothrin, highlights the urgent need for more effective and sustainable vector control strategies. The detection of key genetic mutations, such as V1016G and F1534C in the *Vgsc* gene, underscores the genetic basis of resistance, while the absence of *Ace-1* mutations indicates a potential area for further research in understanding resistance mechanisms.

The temporal and spatial trends observed in resistance patterns, coupled with environmental and demographic factors, reveal the complexity of vector management in Indonesia’s diverse landscape. While existing control methods such as fogging and larviciding remain important, their overuse has accelerated resistance development, reducing their effectiveness and necessitating a shift toward integrated vector management approaches.

Effective resistance management requires a multifaceted strategy that includes routine monitoring of resistance patterns and genetic mutations to inform insecticide selection, rotational or combination use of insecticides to minimize selection pressure, and community engagement to promote environmental management and reduce breeding sites. Strengthening collaborations between policymakers, researchers, and public health practitioners is crucial for designing region-specific, evidence-based interventions.

The findings of this study emphasize the need for continuous surveillance of resistance trends and the genetic evolution of mosquito populations. Addressing these challenges requires prioritizing research on novel control methods, such as biological and genetic approaches, alongside optimizing the current use of chemical insecticides. By implementing a holistic and adaptive vector control strategy, Indonesia can enhance its capacity to combat dengue fever, protect public health, and mitigate the socio-economic burden of this disease.

## AUTHORS’ CONTRIBUTIONS

MRR: Designed and conducted the study. RY, HBN, and MCH: Planned the study and analyzed the results. KCD, BJ, and GMR: Supervised the study. MRR, RY, MCH, and KCD: Corrected and reviewed the manuscript. All authors have read and approved the final manuscript.
